# FOXP4-AS1 Inhibits Papillary Thyroid Carcinoma Proliferation and Migration Through the AKT Signaling Pathway

**DOI:** 10.3389/fonc.2022.900836

**Published:** 2022-06-02

**Authors:** Xue Luo, Qingjun Gao, Tian Zhou, Rui Tang, Yu Zhao, Qifang Zhang, Nanpeng Wang, Hui Ye, Xinghong Chen, Song Chen, Wenli Tang, Daiwei Zhao

**Affiliations:** ^1^Clinical Medical College, Guizhou Medical University, Guiyang, China; ^2^Department of Thyroid Surgery, Affiliated Hospital of Guizhou Medical University, Guiyang, China; ^3^Department of Breast Surgery, Affiliated Hospital of Guizhou Medical University, Guiyang, China; ^4^Department of Thyroid and Breast Surgery, Bijie City First People’s Hospital, Bijie, China; ^5^Department of Thyroid and Breast Surgery, Qian Xi Nan People’s Hospital, Xingyi, China; ^6^Key Laboratory of Endemic and Ethnic Minority Diseases of the Ministry of Education, Guizhou Medical University, Guiyang, China; ^7^Department of Thyroid and Breast Surgery, Jinyang Hospital Affiliated to Guizhou Medical University, Guiyang, China; ^8^Department of Thyroid Surgery, the Second People's Hospital of Guizhou Province, Guiyang, China

**Keywords:** papillary thyroid carcinoma, FOXP4-AS1, FOXP4, AKT signaling pathway, cell proliferation, apoptosis, migration

## Abstract

Papillary thyroid carcinoma, also known as PTC, is one of the commonest malignancies in the endocrine system. Long non-coding RNAs (lncRNAs) in PTC could maintain proliferative signaling, induce therapeutic resistance, activate invasion and migration, and sustain stem cell-like characteristics. In this paper, results showed that lncRNA forkhead box P4 antisense RNA 1 (FOXP4-AS1) is downregulated in PTC tissues and cell lines. Patients in TCGA cohort with a higher FOXP4-AS1 expression showed a higher disease-free interval (DFI) rate, and the expression of FOXP4-AS1 is shown to be linked to the clinical stage, T stage, N stage, and extraglandular invasion condition of the TC patients. FOXP4-AS1 is localized in the cell cytoplasmic domain of PTC cells. Functionally, upregulated FOXP4-AS1 inhibited PTC cell proliferation, apoptosis, and migration, whereas it downregulated FOXP4-AS1-promoted progression of PTC. *In vivo* assay also confirmed the tumor inhibitory effect of FOXP4-AS1 in PTC growth. Mechanism analysis indicated that FOXP4-AS1 can play its functions by regulating the AKT signaling pathway, and AKT inhibitor treatment could attenuate the impact of FOXP4-AS1 on PTC progression. Furthermore, FOXP4-AS1 also negatively regulates the expression of its host gene FOXP4. Collectively, we showed that FOXP4-AS1 inhibited PTC progression although AKT signaling and FOXP4-AS1 plays a tumor-suppressor role in PTC tumorigenesis.

## Introduction

Cancer is one of the leading dangers to human survival worldwide (1–3). Thyroid carcinoma, known as TC, is the most prevalent malignant tumor of the endocrine system (4). There were 586,202 cases of TC worldwide in 2020, which represented 3% of all malignancies, and the death cases were 44,000, which represented 0.4% of all deaths (5). Thyroid carcinoma has several histological types, including follicular thyroid carcinoma, medullary thyroid carcinoma, anaplastic thyroid carcinoma, papillary thyroid carcinoma, squamous cell carcinoma, Hurthle cell carcinoma, and poorly differentiated thyroid carcinoma (6). The incidence of PTC is the highest among all the types of human thyroid carcinoma. BRAF mutation and proto-oncogene tyrosine-protein kinase receptor rearrangements are the most frequent incidence in PTC, which accounts for 40%–60% and 20% of PTC cases, respectively (7). In addition, the promoter sequence of TERT is frequently mutated in PTC patients and contributes to the development of PTC (8). However, the mechanism of PTC is still obscure.

Long non-coding RNA (LncRNA), a common type of non-coding RNA, rarely or does not encode proteins and has more than 200 nucleotides (9). Dysregulation of lncRNAs plays an important role in carcinogenesis or chemotherapy response by modulating various effectors or signaling pathways (10–12). Recently, various lncRNAs in the tumorigenesis of thyroid carcinoma have been well documented, such as LINC00673 (13) and ZFAS1 (14) which play an oncogenic role, while OTUD6B-AS1 (15) and lncRNA-CTC function as tumor suppressor (16). Furthermore, Zhu et al. (6) summarized the abnormal expression of 28 lncRNAs in thyroid carcinoma tumorigenesis and concluded that the functions of lncRNAs were maintaining proliferative signaling and therapeutic resistance, activating migration and invasion, and sustaining stem cell-like characteristics. These reports highlighted that lncRNAs play a vital role in the prognostic and therapeutic in thyroid carcinoma.

lncRNA FOXP4-AS1 is a new cancer-related biomarker that has emerged in recent years, whose roles are diverse in different cancers. It has been reported in several studies with solid proof that FOXP4-AS1 is overexpressed in hepatocellular carcinoma (17), esophageal squamous cell carcinoma (18), prostate cancer (19), nasopharyngeal carcinoma (20, 21), Ewing sarcoma (ES) (22), mantle cell lymphoma (MCL) (23), and osteosarcoma (24), where it promoted cancer progression and played an oncogenic role, and its upregulation usually predicts poor prognosis of these cancers. More importantly, there were reports that indicated that the upregulation of FOXP4-AS1 showed longer overall survival in ovarian cancer (25, 26), suggesting that it could be an efficacious prognostic biomarker.

AKT serine/threonine kinases, which comprise AKT1, AKT2, and AKT3, are essential regulators of cellular metabolism, including glucose uptake, lipid synthesis, and amino acid metabolism (27). Hyperactivation of AKT promotes cell survival, cell cycle progression, and cancer development (27). Numerous downstream effectors of the AKT signaling pathway have been identified, including TSC/mTOR, MDM2, GSK-3, and PFKFB2 (28, 29). However, the upstream modulator of AKT remains to be determined. Since activation of AKT acts as an important contributor for PTC (30–32), studies should be conducted to explore the mechanism of AKT activation during the development of PTC.

However, the role of FOXP4-AS1 in thyroid carcinoma is still obscure. This paper aims at investigating if FOXP4-AS1 does function in TC progression through *in vivo* and *ex vivo* experiments, helping to provide a new perspective for TC treatment.

## Methods and Materials

### Clinical Samples From the Patients

Seventy-two matched papillary thyroid carcinoma (PTC) tissues and paracancerous tissues in total were collected from different stages of PTC patients that all received clinical treatment at the Affiliated Hospital of Guizhou Medical University from August 2019 to September 2021. Liquid nitrogen was used to store the samples. This research was approved by the Research Ethics Committee of Guizhou Medical University, and written informed consents were signed by all participants. The age, clinical stage, N stage, T stage, M stage, and extraglandular invasion condition of the patients were collected and analyzed.

### Survival Analyses in TCGA Database

According to the expression of FOXP4-AS1, TC samples from The Cancer Genome Atlas (TCGA) database were separated into two groups including high group and low group. The prognostic differences between these two were compared using Kaplan–Meier methods.

### Cell Culture

TPC1, K1, and BCPAP cell lines and Nthy-ori3-1 cells were obtained from Guizhou Medical University and cultured in RPMI cell culture medium (HyClone, Logan, UT, USA) with 10% fetal bovine serum (FBS), 100 U/ml penicillin, and 100 mg/ml streptomycin (Solarbio, Beijing, China). The cells were incubated in a cell incubator at 37°C under one atmosphere pressure of air containing 5% CO_2_.

### Fluorescence *In Situ* Hybridization

The experiment was performed according to the manufacturer’s protocol with the Fluorescent *In Situ* Hybridization Kit (Cat no., R11060.7, Guangzhou RiboBio Co., Ltd.). Briefly, cells were fixed by precooling in 4% formaldehyde at 25°C for 30 min, then washed with 1× precooled PBS. After being treated with PBS containing 0.5% Triton X-100 for 8–10 min, cells were pre-hybridized with pre-hybridization buffer at 37°C for 30 min of light aversion. Then, the PTC cells were hybridized in hybridization buffer (FOXP4-AS1 lncRNA probe) in the dark at 37°C overnight. Then, the cells were washed with washing buffer and 1× PBS, and the cells were stained with DAPI buffer. Lastly, the fluorescence signal was read using a fluorescence confocal laser-scanning microscope (Olympus Corporation, Tokyo, Japan) with the excitation at 555 nm.

### Cell Transfection and Cell Infection

As for the transfection experiment, the small interfering RNAs and a negative control for FOXP4-AS1 were designed and synthesized from Huzhou Hippo Biotechnology Co., Ltd. The pcDNA3.1-FOXP4-AS1 plasmid for FOXP4-AS1 overexpression was also purchased from Tianyi-Huiyuan Biotech Co., Ltd. RNAiMAX (Invitrogen, Carlsbad, CA, USA) and Lipofectamine^®^ 3000 transfection reagent (Invitrogen) were applied for transfection of siRNA and plasmid transfection, respectively. For the *in vivo* experiment, the FOXP4-AS1-overexpressed lentivirus was purchased from GeneChem Co., Ltd. (Shanghai, China).

### RNA Extraction and Reverse Transcription-Quantitative Polymerase Chain Reaction

TRIzol reagent (Ambion, Foster City, CA, USA) was utilized for a total RNA isolation, and the ReverTra Ace qPCR-RT Master Mix along with a gDNA Remover (Toyobo, Osaka, Japan) was applied for reverse transcription of total RNA to cDNA. 2x SYBR Green qPCR Master Mix (Low ROX) (Servicebio, Wuhan, China) was used for qPCR by 7500 Fast Dx Real-Time PCR Instruments (Applied Biosystems, Foster City, CA, USA). The primers for PCR were shown as follows: FOXP4-AS1, forward 5′-AAAGGAGACAAAAAGCTCGATGAC-3′ and reverse 5′-TTTTCGCTGCTCTGGAAGATG-3′; FOXP4, forward 5′-ggACACggAgAgTgCAAgTg-3′ and reverse 5′-gTgCTCTgTgTTgAggTgTTT-3′; GADPH, forward 5′-GACTCATGACCACAGTCCATGC-3′ and reverse 5′-AGAGGCAGGGATGATGTTCTG-3′. GADPH was used for normalization, along with the 2^−ΔΔCq^ method which was applied to calculate the expression of genes (33).

### Cell Proliferation Assays

For CCK-8 assay, cells (1 × 10^3^) transfected with siRNAs or plasmids were seeded in a 96-well plate per well and kept accordingly for indicated lengths of time (4, 3, 2, and 1 days), then 10 μl of CCK-8 reagents (Beyotime, Shanghai, China) was added for each well. After being incubated for 2 h, the proliferation of the treated cells each well can be calculated based on absorbance at 450 nm, and the absorbance was detected by an HBS-1096 microplate reader (DeTie, Nanjing, China). Triplicate independent tests were repeated.

For colony formation assay, cells (1 × 10^3^) transfected with siRNAs or plasmids were plated into 6-well plates per well. The medium was refreshed every 3 days. Fourteen days later, the cells were treated with 4% paraformaldehyde for 30 min to fix them and 0.4% crystal violet solution for staining for 0.5 h at 37°C. All the images were captured using a camera system.

### Cell Migration

The migration ability of the indicated cells was studied by transwell assay. The upper chamber with an 8-µm membrane of the 24-well artificial insert was filled with 1 × 10^5^ cells transfected with siRNAs or plasmids in serum-free medium, while the lower chamber was filled with a complete cell culture medium with serum. Twenty-four hours later, the invasive cells with methanol were fixed, and the cells were stained with crystal violet dye, washed with PBS, then counted under a microscope (Nikon, Melville, NY, USA). Triplicate independent tests were repeated.

### Cell Apoptosis and Cell Cycle

For cell apoptosis, cells were transfected with siRNAs or plasmids were cultured for 48 h and harvested through trypsinization. Then, the cells were resuspended with PBS and the concentration of cells adjusted to 1 × 10^6^ cells/ml before staining. After staining the cells with propidium iodide and Annexin V-fluorescein isothiocyanate (Yeasen, Shanghai, China) for an hour on ice light aversion, cell apoptosis could be examined with a flow cytometry system (Beckman, Brea, CA, USA).

For cell-cycle detection, K1 and TPC1 cells transfected with siFOXP4-AS1 for 48 h were synchronized by starving in the G0/G1 phase, then fixed in precooled 75% ethanol and stained with propidium iodide (Yeasen). The DNA content of G0/G1, S, and G2/M phases in the cell cycle was examined through flow cytometry (Beckman). Triplicate independent tests were repeated.

### Western Blot Analysis

Cells were harvested and treated with RIPA buffer (Beyotime). Furthermore, a bicinchoninic acid (BCA) protein assay kit (Thermo, Waltham, MA, USA) was applied to evaluate the concentration of proteins. Thirty micrograms of each sample was separated by 10% SDS-polyacrylamide gel electrophoresis, after which it was transferred to PVDF membranes (Millipore, Burlington, MA, USA). The membrane was blocked with 5% non-fat milk for 1 h at 25°C. Primary antibodies AKT (1:1,000; CST), p-AKT (1:1,000; CST), and GAPDH (1:1,000; CST) were incubated with the membranes overnight at 4°C. Then, TBS was applied to wash the membranes, and secondary antibodies were added to label the protein of interest at room temperature for 2 h. The Western blot bands were quantified by an Odyssey CLx system (LI-COR, Lincoln, NE, USA).

### Tumor Xenograft

In the sum of 10 athymic female nude mice, 8-week-old mice with an average weight at 20 g were divided into two groups. 2,2,2-Tribromoethanol (2%, 200 μl/mouse; Sigma, St. Louis, MO, USA) was used to anesthetize the mice with intraperitoneal injection before implantation. A total of 1 × 10^6^ K1 cells stably transfected with lncRNA FOXP4-AS1 were transferred subcutaneously in mouse flanks. Xenograft size was measured every other day. Then the final volume of xenografts was defined by V = 0.5 × L (length of tumor) × W^2^ (width of tumor). Forty-eight days later, all the treated nude mice were anesthetized to death and engrafted tumors were resected and their weight measured. Our study was approved and conducted by the Institutional Animal Care and Experimental Use Committee of Guizhou Medical University.

### Immunohistochemistry Staining

Four-micrometer sections were formalin-fixed and paraffin-embedded before they were utilized for immunohistochemistry staining. The 4-μm sections were deparaffinized, and antigens were retrieved, rehydrated, and then blocked with 10% goat serum. Then the sections were incubated with the antibodies anti-Ki-67 (1:200, Abcam, Cambridge, MA, USA) or p-AKT (1:200; Abcam) separately overnight at 4°C. The slides were subsequently washed for 3 times before being incubated with secondary antibodies at room temperature for 1 h. Lastly, they were stained with Vulcan Fast Red Chromogen Kit (Biocare Medical, Concord, CA, USA) for 1 h at room temperature.

### Functional Enrichment Analysis

The clusterProfiler package of R software required in this study was utilized for Gene Ontology (GO) function analysis. Basing on the co-expression screening data, the Kyoto Encyclopedia of Genes and Genomes (KEGG) pathway analysis of this study was also performed to dig further information.

### Statistical Analysis

All the experimental data were shown as mean ± standard deviation (SD). GraphPad Prism 8.0 (GraphPad) was utilized for statistical analysis. Paired Students’ t-tests were performed to determine whether there is a difference in the expression of FOXP4-AS1 between PTC tissue and paired control normal tissue. Unpaired Student’s t-test for the two groups was applied to analyze the other differences. One-way ANOVA followed by Tukey’s *post-hoc* test was also applied for comparisons of 3 groups or more. *p* value <0.05 means the results were statistically significant.

## Results

### FOXP4-AS1 Is Low Expressed in Papillary Thyroid Carcinoma and Localized in the Cytoplasm

**Figure 1:**
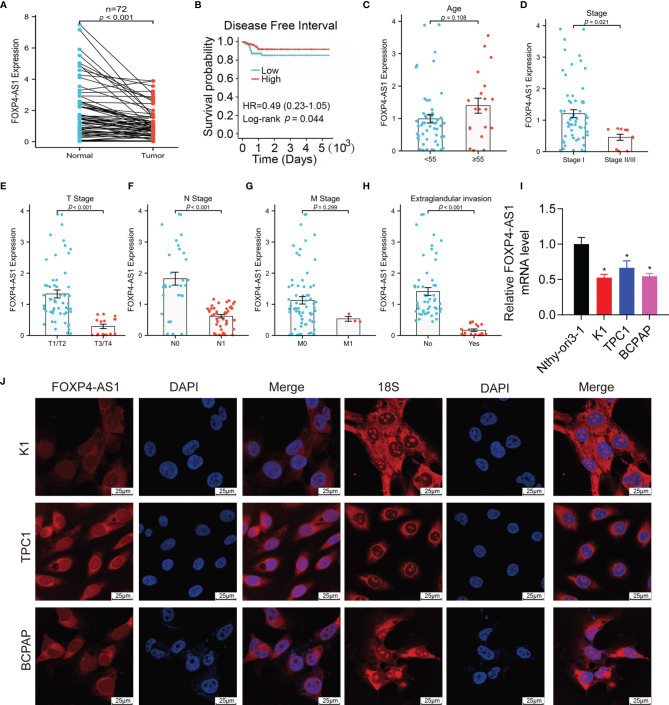
FOXP4-AS1 is low expressed within papillary thyroid carcinoma and localized in the cytoplasm. **(A)** FOXP4-AS1 levels in PTC and adjacent samples. **(B)** The DFI of PTC patients with differential FOXP4-AS1 expression from TCGA database by Kaplan–Meier analysis. **(C)** FOXP4-AS1 levels in patients with PTC above age 55 or below 55. **(D)** FOXP4-AS1 levels in early stage and advanced stage of patients. **(E)** FOXP4-AS1 levels in T3/4 stage and T1/2 stage of patients diagnosed. **(F)** FOXP4-AS1 levels in N1 stage and N0 stages of patients. **(G)** FOXP4-AS1 levels in the M1 stage and M0 stage of patients. **(H)** FOXP4-AS1 levels in patients with extraglandular invasion or without invasion. **(I)** FOXP4-AS1 levels in three cell lines of papillary thyroid carcinoma (K1, BCPAP, and TPC-1) and Nthy-ori3-1 normal cells. **(J)** FOXP4-AS1 was distributed primarily in the cytoplasm of BCPAP, K1, and TPC-1 cells by FISH. Scale bar = 25 μm. *p < 0.05.

We can see from **Figure 1A** that lncRNA FOXP4-AS1 was drastically decreased in papillary thyroid carcinoma clinical samples compared to normal samples by reverse transcription-quantitative polymerase chain reaction (qRT-PCR) assay. All the patients were grouped into low and high groups comparing their FOXP4-AS1 expression value to the median value shown in TCGA database accordingly. Compared with the FOXP4-AS1 low group, the high group had longer PFI (*p* < 0.05; **Figure 1B**), consistent with the reports of Hua et al. (25) and Liao et al. (26). In addition, the expression of FOXP4-AS1 was proved to be linked with the clinical stage, T stage, N stage, and extraglandular invasion condition of the PTC patients (*p* < 0.05, **Figures 1C–H**). Furthermore, lncRNA FOXP4-AS1 was downregulated in three thyroid carcinoma cell lines compared to normal thyroid cells (**Figure 1I**). Besides, the fluorescence *in situ* hybridization (FISH) results showed that FOXP4-AS1 distributes predominantly in the PTC cell cytoplasm (**Figure 1J**), which was consistent with Xiong et al.’s report (22).

### FOXP4-AS1 Inhibits Papillary Thyroid Carcinoma Cell Proliferation

**Figure 2:**
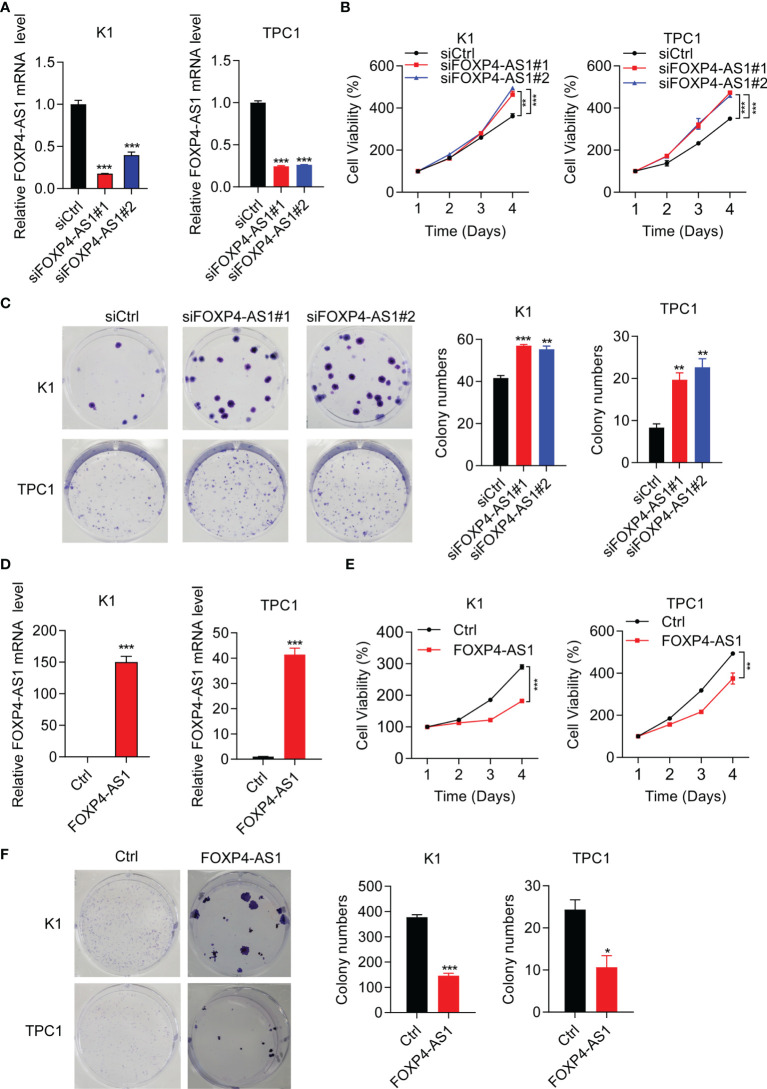
FOXP4-AS1 inhibits papillary thyroid carcinoma cell proliferation. **(A)** Knockdown efficiency examined in K1 and TPC1 cell lines of siFOXP4-AS1. **(B)** Cell viability of two PTC lines transfected with siFOXP4-AS1 was determined by CCK-8 assay. **(C)** Cell colony numbers of two PTC lines transfected with siFOXP4-AS1 were recorded by cell colony formation assay. **(D)** Overexpression efficiency of FOXP4-AS1 in K1 and TPC1 cells. **(E)** Cell viability of two PTC cell lines transfected using FOXP4-AS1 was determined by CCK-8 assay. **(F)** Cell colony numbers of two PTC cell lines transfected using FOXP4-AS1 was calculated by cell colony formation assay. *p < 0.05, **p < 0.01, ***p < 0.001.

Short interference siRNAs (si-FOXP4-AS1) were transferred into K1 and TPC1 cells to knock down FOXP4-AS1, and the efficiency was verified by RT-qPCR (**Figure 2A**). The cell viabilities after FOXP4-AS1 knockdown were increased significantly compared with siCtrl in two PTC cell lines (**Figure 2B**). Meanwhile, knockdown of FOXP4-AS1 also increased cell colony numbers in comparison with siCtrl (**Figure 2C**). Conversely, the efficiency of overexpression of FOXP4-AS1 by the pcDNA3.1-FOXP4-AS1 plasmid in three thyroid carcinoma cells was also verified by RT-qPCR (**Figure 2D**), and overexpression of FOXP4-AS1 remarkably inhibited growth (**Figures 2E, F****)**. These results showed that FOXP4-AS1 can inhibit thyroid carcinoma cell proliferation.

### FOXP4-AS1 Induces Apoptosis of Papillary Thyroid Carcinoma Cells, but Has Little Effect on the Cell Cycle

**Figure 3:**
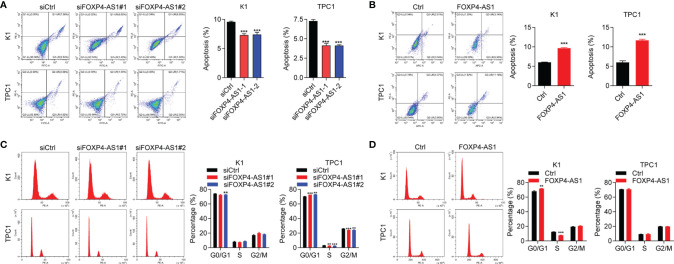
The effect of FOXP4-AS1 on apoptosis and cell cycle of papillary thyroid carcinoma cells. **(A)** The apoptotic rate of TPC1 and K1 cell lines transfected with FOXP4-AS1 was analyzed by flow cytometry. **(B)** The apoptotic rate of K1 and TPC1 cell lines following FOXP4-AS1 overexpression was analyzed by flow cytometry experiment. **(C)** Cell cycle distribution ratio of K1 and TPC1 cell lines transfected with FOXP4-AS1 and corresponding control was analyzed by flow cytometry. **(D)** Cell cycle distribution of K1 as well as TPC1 cell lines following FOXP4-AS1 overexpression was examined by flow cytometry. *p < 0.01, ***p < 0.001.

Our flow cytometry experiments showed that the cell apoptosis ratio was inhibited after FOXP4-AS1 knockdown in K1 and TPC1 cells (**Figure 3A**), while it was promoted with FOXP4-AS1 overexpression (**Figure 3B**). The flow cytometry experiment was also applied for the comparison of the cell distribution ratio of the cell cycle between FOXP4-AS1 downregulation or overexpression cells and vector cells. The results showed no significant change of each stage in the cell cycle during FOXP4-AS1 knockdown or FOXP4-AS1 overexpression (**Figures 3C, D****)**. These results indicated that FOXP4-AS1 could induce PTC cell apoptosis, but has little effect on the cell cycle.

### FOXP4-AS1 Inhibits Papillary Thyroid Carcinoma Cell Migration

**Figure 4:**
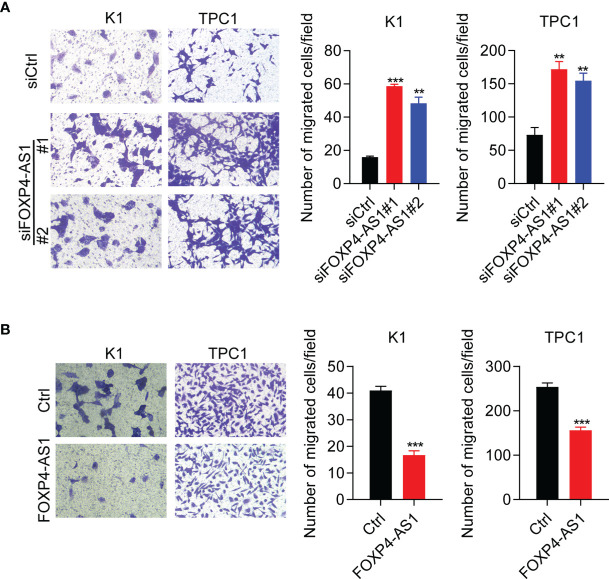
FOXP4-AS1 inhibits papillary thyroid carcinoma cell migration. **(A)** Transwell assay results of the migratory capability of TPC1 and K1 cell lines transfected with siFOXP4-AS1. **(B)** Transwell assay results of the migratory capability of TPC1 and K1 cells following FOXP4-AS1 overexpression. **p < 0.01, ***p < 0.001.

Furthermore, transwell assay results suggested that cell migration was significantly promoted in TPC and K1 cells after FOXP4-AS1 knockdown (**Figure 4A**), whereas it was greatly suppressed following FOXP4-AS1 overexpression (**Figure 4B**). These results showed that FOXP4-AS1 inhibits papillary thyroid carcinoma cell migration.

### FOXP4-AS1 Inhibits Tumorigenesis of Papillary Thyroid Carcinoma *In Vivo*

**Figure 5:**
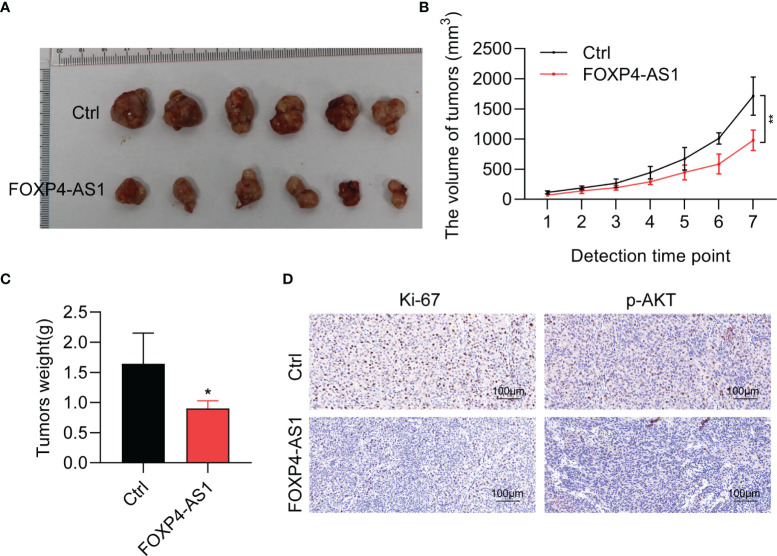
FOXP4-AS1 inhibits tumorigenesis of papillary thyroid carcinoma in nude mice. **(A)** K1 cells, FOXP4-AS1-overexpressed K1 cells, and control K1 cells were injected in nude mouse flank to observe the tumor growth. **(B, C)** Statistical analysis of the **(B)** tumor volume and **(C)** tumor weight between two groups. **(D)** Ki-67 and p-AKT protein expression in xenografts with FOXP4-AS1 overexpression or control groups by IHC. *p < 0.05; **p < 0.01.

To detect the physiological function of FOXP4-AS1 *in vivo*, a stable FOXP4-AS1-overexpressed K1 cell was constructed. Tumor xenograft assay results showed that FOXP4-AS1 overexpression could significantly impair the tumor size, tumor volume, and tumor weight (**Figures 5A–C**), which was consistent with our analysis *in vitro*. Moreover, the expression of tumor marker Ki-67 and a signaling molecular p-AKT was decreased in nude mice with FOXP4-AS1 overexpression by immunohistochemical staining (**Figure 5D**). These findings indicated that FOXP4-AS1 inhibits tumorigenesis of papillary thyroid carcinoma *in vivo*.

### FOXP4-AS1 Can Reverse Regulate FOXP4 Expression and Inhibit the Activity of the Akt Signaling Pathway

**Figure 6:**
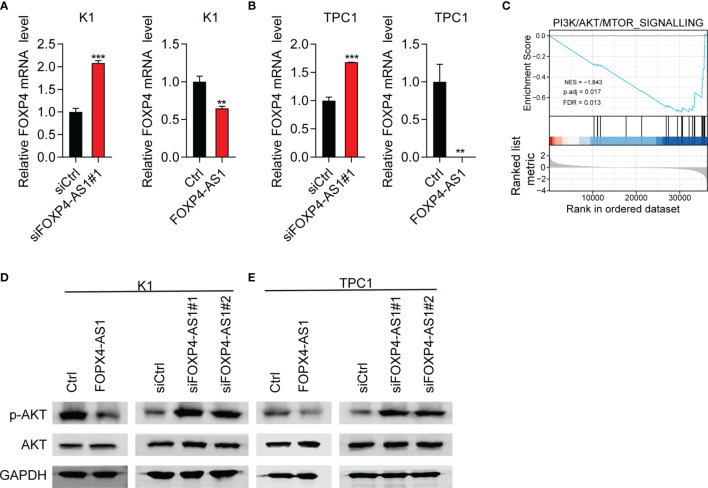
FOXP4-AS1 can reverse-regulate FOXP4 expression and inhibit the activity of p-Akt. **(A, B)** The mRNA level of FOXP4 in **(A)** K1cell and **(B)** TPC-1 cell FOXP4-AS1 knockdown or overexpression was detected by RT-qPCR. **(C)** PI3K/AKT signaling pathway was enriched according to GSEA analysis. **(D, E)** The protein level of p-AKT in **(D)** K1 and **(E)** TPC-1 cells after knockdown of FOXP4-AS1 or overexpression was detected using western blot method. **p < 0.01, ***p < 0.001.

FOXP4 was a predicted target gene of FOXP4-AS1 (25). Therefore, we suggested that FOXP4-AS1 might play its function through regulating FOXP4 expression. The expression of FOXP4 mRNA was decreased significantly in K1 and TPC1 cells following FOXP4-AS1 overexpression as expected, while it was significantly increased after FOXP4-AS1 knockdown in K1 and TPC1 cells (**Figures 6A, B****)**.

To further study the role of FOXP4-AS1 in thyroid carcinoma, we analyzed the FOXP4-AS1-associated signaling pathway based on TCGA database. GSEA analyses showed that enrichment of the PI3K/AKT signaling pathway was significant after FOXP4-AS1 knockdown (**Figure 6C**). The PI3K/AKT pathway appears to be one of the major signaling pathways that contribute to tumorigenesis of thyroid carcinoma (6). The expression level of p-AKT was impaired significantly in K1 and TPC1 cells following FOXP4-AS1 overexpression by Western blotting, whereas it increased after FOXP4-AS1 knockdown (**Figures 6D, E****)**. These results suggested that FOXP4-AS1 can negatively regulate FOXP4 expression and inhibit the activity of the Akt signaling pathway in thyroid carcinoma cell lines.

### AKT Inhibitor Can Partially Reverse the Promoting Effect of siFOXP4-AS1 on PTC Cell Lines

**Figure 7:**
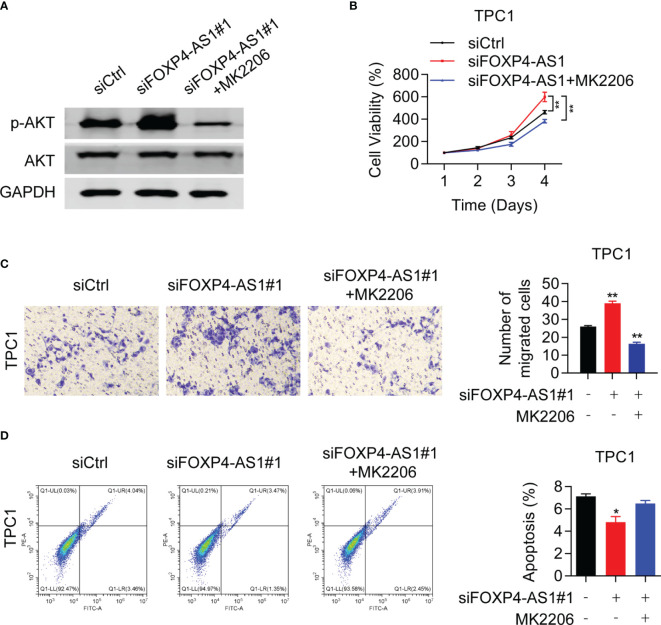
Akt inhibitor can reverse the promoting effect of siFOXP4-AS1 on papillary thyroid cancer cell lines. **(A)** The inhibitory efficiency of MK2206 to p-AKT. **(B)** The effect of siFOXP4-AS1 on PTC cell viability was attenuated by AKT inhibitor MK2206. **(C)** The effecting of siFOXP4-AS1 on PTC cell colony numbers was attenuated by MK2206. **(D)** The effect of MK2206 on siFOXP4-AS1-mediated apoptosis. *p < 0.05, **p < 0.01.

To confirm whether FOXP4-AS1 inhibited the PTC progression *via* AKT signaling, rescue assays were conducted. Western blot results proved that the p-AKT inhibitor MK2206 effectively inhibited the activation of AKT signaling in TPC1 cells (**Figure 7A**), which greatly suppressed the protein level of p-AKT. CCK-8 assay demonstrated that MK2206 attenuated the impact of siFOXP4-AS1 on TPC1 cell viabilities (**Figure 7B**), and the colony formation of cells (**Figure 7C**) as well as the cell apoptosis rate (**Figure 7D**) was partially reversed in TPC1 cells by colony formation assay as well as flow cytometry experiments. These solid results confirmed that FOXP4-AS1 inhibited PTC growth and migration through regulating the AKT signaling pathway.

## Discussion

Currently, emerging reports indicate the functions of lncRNAs regarding the occurrence of thyroid carcinoma (TC). For instance, lncRNA LINC00673 possesses the function of inducing cell proliferation, migration, and metastasis as well as epithelial-mesenchymal transition in TC by regulating Kruppel-like factor 2 (KLF2) (13). By contrast, lncRNA OTUD6B-AS1 possesses the function of inhibiting the cell viability, tumor migration, and tumor invasion of TC *via* miR-21 along with miR-183-5p (15). LncRNA FOXP4-AS1 was shown to be associated with many cancers (17–19). In this piece, we investigated that the expression of FOXP4-AS1 was rather low in PTC cells as well as the tissues. Compared with the low group, the patients in the high FOXP4-AS1 group from TCGA had a longer disease-free interval. Furthermore, FOXP4-AS1 expression was related to the clinical stage, N stage, T stage, and extraglandular invasion condition of PTC patients. This suggests that the lower the expression of lncRNA FOXP4-AS1, the more severe the disease tends to be.

Previous studies have implied that FOXP4-AS1 may function as an oncogene in a variety of tumors. FOXP4-AS1, expressed highly in colorectal cancer (CRC) tissue, can promote the colorectal cancer cell proliferation, inhibit apoptosis, and downregulate the tumor-suppressor gene expression including P15, P21, P27, and KLF2, which are positively related to tumor size and pathological stages. The FOXP4-AS1 expression level is positively correlated with tumor malignancy (34). In gastric cancer and osteosarcoma, studies have found that its high expression of FOXP4-AS1 was linked to late clinical stage and poor prognosis (24, 35). Similarly, in hepatocellular carcinoma (HCC), FOXP4-AS1 can also accelerate HCC progression by recruiting EZH2 to suppress ZC3H12D expression (36). However, in our study, the results suggest that FOXP4-AS1 could inhibit proliferating activities and migration of cells and induce cell apoptosis, therefore indicating the suppressor function of FOXP4-AS1 in PTC. Similar results were obtained in FOXP4-AS1 over expressed nude mice. These results illustrated that FOXP4-AS1 functioned as an emerging tumor suppressor in PTC progression. Hence, we speculate that the physiological function of FOXP4-AS1 in tumor is microenvironment dependent. Moreover, this is the first study to discover that FOXP4-AS1 possesses the function of a tumor-suppressor gene to the best of our knowledge.

It was researched that FOXP4-AS1 can not only modulate the epigenetic modification of the genome by recruiting epigenetic modification proteins in the nucleus but also affect the regulation of miRNAs on target genes by acting as a molecular sponge for miRNAs in the cytoplasm, in tumor studies. We went further to detect its subcellular localization and found that it localized at the cytoplasm of thyroid cells consistent with a previous report (22). Therefore, we speculate that FOXP4-AS1 functions as a tumor suppressor in PTC through two possible ways: acting as a miRNA sponge or regulating the stability of its binding proteins. Further studies are required to clarify the mechanism. FOXP4 is the host gene of FOXP4-AS1 (19), belonging to the human forkhead-box (FOX) gene family, which plays crucial roles in tumor oncogenesis and cell cycle regulation (37). Li et al. showed that FOXP4-AS1 regulates the expression of FOXP4 positively by regulating miR-3184-5p in esophageal squamous cell carcinoma (ESCC) cells (18). Moreover, in Niu et al.’s study, they found a positive relation between FOXP4 and FOXP4-AS1 in ESCC (38). A positive association between FOXP4-AS1 and FOXP4 was also found in ovarian cancer (OC) tissues (25). In contrast, we found that the FOXP4 expression was interestingly negatively regulated by the gene FOXP4-AS1. Therefore, we can infer that when FOXP4-AS1 acts as an oncogene, it positively regulates the expression of FOXP4, whereas if FOXP4-AS1 is a tumor suppressor, it negatively regulates the expression of FOXP4.

**Figure 8:**
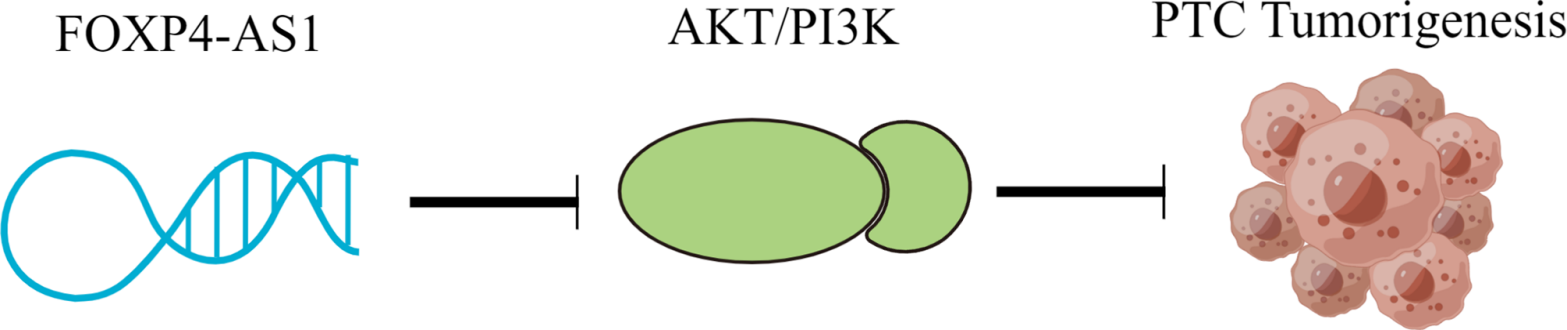
Role of the FOXP4-AS1/PI3K/AKT signaling pathway in PTC.

The PI3K/AKT/mTOR signaling pathway was featured strongly in regulating cellular metabolism, proliferation, survival, and apoptosis (29). Hyperactivation of the PI3K/AKT/mTOR signaling pathway is proved to contribute to the development of various cancers, including thyroid carcinoma (6, 27, 39). Although the activation of PI3K/AKT/mTOR signaling is observed in almost all PTC patients, the upstream regulator of this signaling is still to be determined. In this research, our results indicated that the p-AKT protein level was greatly decreased in FOXP4-AS1 over expressed nude mice, and KEGG pathway analyses showed the high enrichment of the PI3K/AKT signaling pathway in FOXP4-AS1 co-expression pathways. Therefore, whether FOXP4-AS1 inhibited PTC growth through regulating the AKT signaling pathway should be addressed. Consistent with the above previous studies, FOXP4-AS1 overexpression suppressed the phosphorylation levels of AKT, whereas FOXP4-AS1 knockdown had the opposite effect on p-AKT. *In vivo*, FOXP4-AS1 overexpression resulted in reduced AKT phosphorylation levels during PTC tumor development, suggesting that FOXP4-AS1 inhibition of AKT activity is important for its tumor-suppressive role. Moreover, the AKT inhibitor could partially impair the impact of siFOXP4-AS1 on PTC cell proliferation, migration, and apoptosis, indicating that FOXP4-AS1 could modulate PTC progression by the AKT signaling pathway. Collectively, these results identify that FOXP4-AS1 negatively regulates AKT activity and the FOXP4-AS1/AKT signaling axis is essential for PTC progression (**Figure 8**).

In summary, our study illustrated that FOXP4-AS1 was downregulated in PTC. FOXP4-AS1 could inhibit PTC cell proliferation, migration, and apoptosis through the AKT signaling pathway, and it can also reverse the expression of its host gene FOXP4. Thus, this study revealed FOXP4-AS1’s function as a tumor suppressor in PTC tumorigenesis. All our findings may provide a new idea for exploring therapeutic biomarkers for PTC.

## Data Availability Statement

The datasets presented in this study can be found in online repositories. The names of the repository/repositories and accession number(s) can be found in the article/supplementary material.

## Ethics Statement

The studies involving human participants were reviewed and approved by the Ethics Committee of Guizhou Medical University. The patients/participants provided their written informed consent to participate in this study. The animal study was reviewed and approved by Animal Experimental Ethics Inspection Form Guizhou Medical University.

## Author Contributions

All authors listed have made a substantial, direct, and intellectual contribution to the work and approved it for publication.

## Funding

The study was supported by the National Natural Science Foundation of China (No. 81860478).

## Conflict of Interest

The authors declare that the research was conducted in the absence of any commercial or financial relationships that could be construed as a potential conflict of interest.

## Publisher’s Note

All claims expressed in this article are solely those of the authors and do not necessarily represent those of their affiliated organizations, or those of the publisher, the editors and the reviewers. Any product that may be evaluated in this article, or claim that may be made by its manufacturer, is not guaranteed or endorsed by the publisher.
